# Thermal Convection of Nanoliquid in a Double-Connected Chamber

**DOI:** 10.3390/nano10030588

**Published:** 2020-03-23

**Authors:** Ioan Pop, Mikhail A. Sheremet, Teodor Groşan

**Affiliations:** 1 Department of Applied Mathematics, Babeş-Bolyai University, Cluj-Napoca 400084, Romania; trgosan@math.ubbcluj.ro; 2 Laboratory on Convective Heat and Mass Transfer, Tomsk State University, Tomsk 634050, Russia; sheremet@math.tsu.ru

**Keywords:** nanoparticles, natural convection, conjugate heat transfer, heat-conducting block

## Abstract

Thermogravitational convective thermal transmission, inside a square differentially-heated chamber with a nanoliquid, has been examined in the presence of internal adiabatic or a thermally-conducting solid body. A single-phase nanoliquid approach is employed, based on the experimentally-extracted relations for nanofluid heat conductivity and dynamic viscosity. The governing equations have been written using non-primitive parameters such as stream function and vorticity. Such approach allows a decrease in computational time due to a reduction of equation numbers. One of the main challenges in such a technique is a determining the stream function magnitude at the inner body walls. A solution of this problem has been described in detail in this paper. Computational scrutinizing has been performed by employing the finite difference technique. The mesh sensitivity analysis and comparison with theoretical and experimental results of other researchers have been included. An influence of the Rayleigh number, nanoparticles concentration, internal block size, heat conductivity ratio and non-dimensional time on nanofluid motion and energy transport has been studied.

## 1. Introduction

The investigation of heat-driven liquid motion and energy transfer in chambers is a significant subject due to its huge applications in practice, including thermal collectors, thermal exchangers, microelectronic gears, phenomena within buildings and many others [1,2]. Now there are many published papers and books on thermal convection within chambers. For example, a great review on thermal convective energy transport can be found in [3], where the complex nature of free convection phenomena in enclosures is discussed. An analysis is presented for two-dimensional (2D) convection flow, subjected by the buoyancy force on the liquid in a domain. Major efforts are directed to the various motion modes that can happen and the energy transport across the liquid area between the two flat parallel vertical surfaces. The rectangular chamber topic is considered as the most wide-spread benchmark task in numerical liquid flow and energy transport literature. This not only arises from theoretical benchmark data, but also for its practical applications where both, the liquid circulation and energy transport are within the chamber.

Nanoliquids play an essential role in energy transport applications with promising parameters that can be managed. Nanosuspensions have significant properties that allow performing analysis by many scientists to model new thermal systems for various practical applications. Mono-nanoliquids, created using a single sort of nanoadditives, have essential benefits due to defining nanoliquids, which is the combination of tiny-sized solid particles in conventional fluids, many experimental and numerical investigations have demonstrated use of these kind of liquids. Choi [4] studied the process of suspending nano-sized solid particles in the host liquid and considered this liquid as a nanoliquid. The most essential feature of nanoliquid is that coagulation can be stopped. The homogeneous distribution of solid particles and introduction of necessary surfactant can stop the formation of set of conglutinated particles (Babu et al. [5]). Many investigations about energy transport intensification using nanoliquids have been published. Papers and books on energy transport in nanoliquids can be found in [6,7,8,9,10,11,12,13,14,15,16,17,18,19,20,21].

The objective of this research is to computationally scrutinize the free convective energy transport of nanofluid in a differentially-heated chamber, having internal blocks, using the mathematical nanofluid model based, on single-phase nanofluid approach. We have described, in detail, a technique for the definition of stream function magnitude at an inner body surface in a double-connected domain. It should be noted that the present paper deals with an analysis of heat transfer performance of nanoliquid in a cavity with internal solid block. Such problem can be found in the case of optimization of the electronics cooling system, where the electronic cabinet includes different solid blocks. In the case of simple geometry an investigation of the internal body size and thermal conductivity has been conducted for various nanoadditives concentration. Moreover, nowadays scientists use primitive variables for analysis to solve convective heat transfer problems, within multi-connected domains, and as a result, there are have been no problems with the definition of the velocity at internal solid block surface. In the case of non-primitive variables, such as stream function and vorticity, it is necessary to develop a special algorithm for definition of the stream function at internal solid block surface, and such algorithm has been developed in the present study. It is well-known that employing the non-primitive variables reduces the number of governing equations, and as a result, the computational time. By using this developed method, it is possible to conduct an effective analysis of the velocity and temperature fields within electronic cabinet having adiabatic of heat-conducting solid block. Different structure of the inner body can be used for an intensification of the convective heat transfer under the influence of nano-sized particles volume fraction.

## 2. Mathematical Model

**Figure 1:**
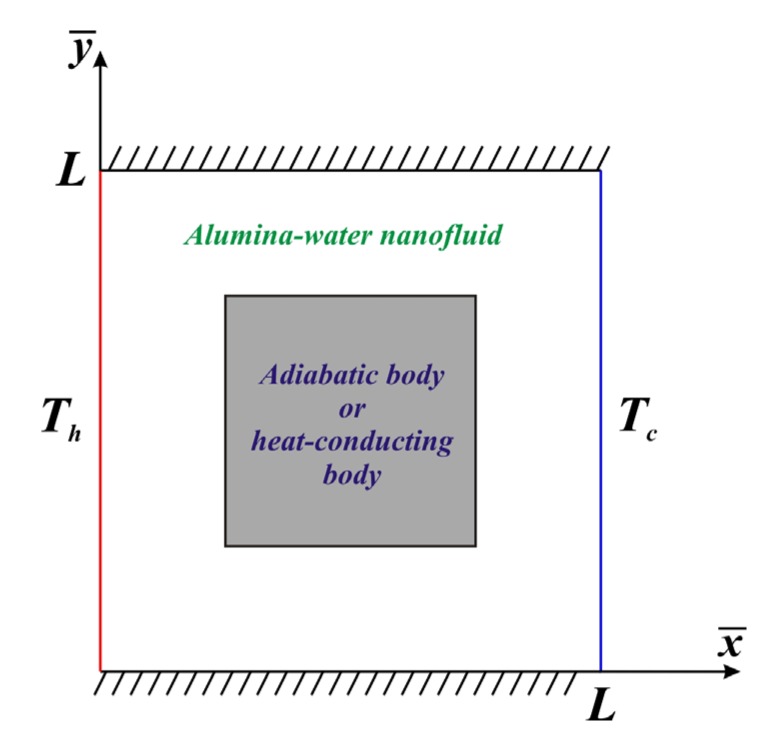
Sketch of the problem.

It is important to consider the transient natural convective heat transport in 2D differentially-heated nanoliquid chamber of height *L* in the presence of inner adiabatic or thermally-conducting block. It is supposed that the left border is hot with temperature *T_h_*, whilst the right border is cold with temperature *T_c_*. The horizontal boundaries are thermally insulated $\left(\partial T/\partial \bar{y}\right)=0$ (Figure 1). For the description of the transport processes within a nanoliquid the single-phase model with effective parameters is used. Such approach illustrates that the nanoadditives are uniformly included in the host liquid. Moreover, using the single-phase model with experimentally-based correlations for physical properties is more effective in comparison with two-phase nanoliquid models and experimental data [22,23].

Using the Boussinesq approach, the basic equations managing these phenomena can be formulated in dimensional form as [24]:

(1) $$\frac{\partial \bar{u}}{\partial \bar{x}}+\frac{\partial \bar{v}}{\partial \bar{y}}=0$$

(2) $$\rho_{nf}\left(\frac{\partial \bar{u}}{\partial t}+\bar{u}\frac{\partial \bar{u}}{\partial \bar{x}}+\bar{v}\frac{\partial \bar{u}}{\partial \bar{y}}\right)=-\frac{\partial \bar{p}}{\partial \bar{x}}+\mu_{nf}\left(\frac{\partial^{2}\bar{u}}{\partial {\bar{x}}^{2}}+\frac{\partial^{2}\bar{u}}{\partial {\bar{y}}^{2}}\right)$$

(3) $$\rho_{nf}\left(\frac{\partial \bar{v}}{\partial t}+\bar{u}\frac{\partial \bar{v}}{\partial \bar{x}}+\bar{v}\frac{\partial \bar{v}}{\partial \bar{y}}\right)=-\frac{\partial \bar{p}}{\partial \bar{y}}+\mu_{nf}\left(\frac{\partial^{2}\bar{v}}{\partial {\bar{x}}^{2}}+\frac{\partial^{2}\bar{v}}{\partial {\bar{y}}^{2}}\right)+{\left(\rho \beta \right)}_{nf}g\left(T-T_{c}\right)$$

(4) $${\left(\rho c\right)}_{nf}\left(\frac{\partial T}{\partial t}+\bar{u}\frac{\partial T}{\partial \bar{x}}+\bar{v}\frac{\partial T}{\partial \bar{y}}\right)=k_{nf}\left(\frac{\partial^{2}T}{\partial {\bar{x}}^{2}}+\frac{\partial^{2}T}{\partial {\bar{y}}^{2}}\right).$$

In the case of internal heat-conducting block an additional heat conduction equation has been included in the following form [25]:

(5) $${\left(\rho c\right)}_{s}\frac{\partial T}{\partial t}=k_{s}\left(\frac{\partial^{2}T}{\partial {\bar{x}}^{2}}+\frac{\partial^{2}T}{\partial {\bar{y}}^{2}}\right).$$

Additional relations for the considered problem are:

(6) $$\begin{array}{l} t=0:\;\bar{u}=\bar{v}=0,\;T=T_{c}\;\mathrm{at}\;0\leq \bar{x}\leq L,\;0\leq \bar{y}\leq L;\\ t>0:\;\bar{u}=\bar{v}=0,\;T=T_{h}\;\mathrm{at}\;\bar{x}=0,\;0\leq \bar{y}\leq L;\\ \bar{u}=\bar{v}=0,\;T=T_{c}\;\mathrm{at}\;\bar{x}=L,\;0\leq \bar{y}\leq L;\\ \bar{u}=\bar{v}=0,\;\frac{\partial T}{\partial \bar{y}}=0\;\mathrm{at}\;\bar{y}=0,\;L,\;0\leq \bar{x}\leq L\\ \bar{u}=\bar{v}=0,\;\frac{\partial T}{\partial \bar{n}}=0\;\mathrm{at}\;\mathrm{internal}\;\mathrm{adiabatic}\;\mathrm{block}\\ \left\{ \begin{array}{l} T_{nf}=T_{s}\\ k_{nf}\frac{\partial T_{nf}}{\partial \bar{n}}=k_{s}\frac{\partial T_{s}}{\partial \bar{n}} \end{array}\right.\;\mathrm{at}\;\mathrm{internal}\;\text{heat-conducting block} \end{array}$$

The nanoliquid properties are [24,26]:

(7) $$\rho_{nf}=\left(1-\phi \right)\rho_{f}+\phi \rho_{p}$$

(8) $${\left(\rho c\right)}_{nf}=\left(1-\phi \right){\left(\rho c\right)}_{f}+\phi {\left(\rho c\right)}_{p}$$

(9) $${\left(\rho \beta \right)}_{nf}=\left(1-\phi \right){\left(\rho \beta \right)}_{f}+\phi {\left(\rho \beta \right)}_{p}.$$

The nanoliquid thermal conductivity was defined using the experimental data [27]:

(10) $$k_{nf}=k_{f}\left(1+2.944\phi +19.672\phi^{2}\right).$$

in the case of nanoliquid dynamic viscosity the following correlation was used [27]:

(11) $$\mu_{nf}=\mu_{f}\left(1+4.93\phi +222.4\phi^{2}\right).$$

These correlations are valid for 1%≤ϕ≤4%.

The non-dimensional parameters are used,

(12) $$\begin{array}{c} x=\bar{x}/L,\;y=\bar{y}/L,\;\tau =V_{0}t/L,\;u=\bar{u}/V_{0},\;v=\bar{v}/V_{0},\;\theta =\left(T-T_{c}\right)/\left(T_{h}-T_{c}\right),\\ \psi =\bar{\psi }/\left(V_{0}L\right),\;\omega =\bar{\omega }L/V_{0},\;V_{0}=\sqrt{g\beta \left(T_{h}-T_{c}\right)L} \end{array}$$

and employing the non-dimensional stream function *ψ*, which is determined as u=∂ψ/∂y and v=−∂ψ/∂x, as well as non-dimensional vorticity *ω* (ω=∂v/∂x−∂u/∂y) we obtain:

(13) $$\frac{\partial^{2}\psi }{\partial x^{2}}+\frac{\partial^{2}\psi }{\partial y^{2}}=-\omega$$

(14) $$\frac{\partial \omega }{\partial \tau }+u\frac{\partial \omega }{\partial x}+v\frac{\partial \omega }{\partial y}=\frac{\mu_{nf}}{\mu_{f}}\frac{\rho_{f}}{\rho_{nf}}\sqrt{\frac{Pr}{Ra}}\left(\frac{\partial^{2}\omega }{\partial x^{2}}+\frac{\partial^{2}\omega }{\partial y^{2}}\right)+\frac{{\left(\rho \beta \right)}_{nf}}{\rho_{nf}\beta_{f}}\frac{\partial \theta }{\partial x}$$

(15) $$\frac{\partial \theta }{\partial \tau }+u\frac{\partial \theta }{\partial x}+v\frac{\partial \theta }{\partial y}=\frac{1}{\sqrt{Ra\cdot Pr}}\frac{k_{nf}}{k_{f}}\frac{{\left(\rho c_{p}\right)}_{f}}{{\left(\rho c_{p}\right)}_{nf}}\left(\frac{\partial^{2}\theta }{\partial x^{2}}+\frac{\partial^{2}\theta }{\partial y^{2}}\right).$$

In the case of internal heat-conducting solid body we should add to the previous system of equations the following non-dimensional heat conduction equation:

(16) $$\frac{\partial \theta }{\partial \tau }=\frac{1}{\sqrt{Ra\cdot Pr}}\frac{k_{s}}{k_{f}}\frac{{\left(\rho c\right)}_{f}}{{\left(\rho c\right)}_{s}}\left(\frac{\partial^{2}\theta_{s}}{\partial x^{2}}+\frac{\partial^{2}\theta_{s}}{\partial y^{2}}\right).$$

Initial and boundary relations for the obtained equations are:

(17) $$\begin{array}{l} \tau =0:\;\psi =0,\;\omega =0,\;\theta =0.5\;\mathrm{at}\;0\leq x\leq 1,\;0\leq y\leq 1;\\ \tau >0:\;\psi =0,\;\frac{\partial \psi }{\partial x}=0,\;\theta =1\;\mathrm{at}\;x=0,\;0\leq y\leq 1;\\ \psi =0,\;\frac{\partial \psi }{\partial x}=0,\;\theta =0\;\mathrm{at}\;x=1,\;0\leq y\leq 1;\\ \psi =0,\;\frac{\partial \psi }{\partial y}=0,\;\frac{\partial \theta }{\partial y}=0\;\mathrm{at}\;y=0,\;1,\;0\leq x\leq 1\\ \psi =\gamma ,\;\frac{\partial \psi }{\partial n}=0,\;\frac{\partial \theta }{\partial n}=0\;\mathrm{at}\;\mathrm{internal}\;\mathrm{adiabatic}\;\mathrm{block}\\ \psi =\gamma ,\;\frac{\partial \psi }{\partial n}=0,\;\left\{ \begin{array}{l} \theta_{nf}=\theta_{s}\\ \frac{k_{nf}}{k_{s}}\frac{\partial \theta_{nf}}{\partial n}=\frac{\partial \theta_{s}}{\partial n} \end{array}\right.\;\mathrm{at}\;\mathrm{internal}\;\text{heat-conducting solid block} \end{array}$$

(18) $$\tau >0:\;\psi =\gamma ,\;\frac{\partial \psi }{\partial n}=0,\;\frac{\partial \theta }{\partial n}=0\;\mathrm{at}\;\mathrm{internal}\;\mathrm{adiabatic}\;\mathrm{block}$$

(19) $$\tau >0:\;\psi =\gamma ,\;\frac{\partial \psi }{\partial n}=0,\;\left\{ \begin{array}{l} \theta_{nf}=\theta_{s}\\ \frac{k_{nf}}{k_{s}}\frac{\partial \theta_{nf}}{\partial n}=\frac{\partial \theta_{s}}{\partial n} \end{array}\right.\;\mathrm{at}\;\text{internal heat-conducting}\;\mathrm{solid}\;\mathrm{block}$$

Here $Ra=\frac{g{\left(\rho \beta \right)}_{f}{\left(\rho c_{p}\right)}_{f}\left(T_{h}-T_{c}\right)L^{3}}{\mu_{f}k_{f}}$ is the Rayleigh number, $Pr=\frac{{\left(\mu c_{p}\right)}_{f}}{k_{f}}$ is the Prandtl number, additional factors in vorticity Equation (14) are $\frac{\mu_{nf}}{\mu_{f}}\frac{\rho_{f}}{\rho_{nf}}=\frac{1+4.93\phi +222.4\phi^{2}}{1-\phi +\phi \rho_{p}/\rho_{f}}$, $\frac{{\left(\rho \beta \right)}_{nf}}{\rho_{nf}\beta_{f}}=\frac{1-\phi +\phi {\left(\rho \beta \right)}_{p}/{\left(\rho \beta \right)}_{f}}{1-\phi +\phi \rho_{p}/\rho_{f}}$, a factor in energy Equation (15) is $\frac{k_{nf}}{k_{f}}\frac{{\left(\rho c\right)}_{f}}{{\left(\rho c\right)}_{nf}}=\frac{1+2.944\phi +19.672\phi^{2}}{1-\phi +\phi {\left(\rho c\right)}_{p}/{\left(\rho c\right)}_{f}}$, while $\frac{k_{nf}}{k_{s}}=\left(1+2.944\phi +19.672\phi^{2}\right)K$ is a factor for temperature boundary condition of forth kind in Equation (19). Here $K=k_{f}/k_{s}$ is the heat conductivity ratio.

As a result, the boundary-value problem of thermogravitational convection in a double-connected domain having isolated internal body includes Equations (13)–(15), with conditions (17) and (18), while for the internal thermally-conducting solid body, Equations (13)–(16) need to be solved with additional relations (17) and (19).

For description of the overall energy transfer the local Nusselt number at heated wall was defined as,

(20) $$Nu=-\frac{k_{nf}}{k_{f}}{\frac{\partial \theta }{\partial x}|}_{x=0}$$

and the average Nusselt number $\left(\bar{Nu}\right)$ can be considered as:

(21) $$\bar{Nu}=\int_{0}^{1}Nu\;dy.$$

## 3. Numerical Technique

The written governing Equations (13)–(16), with additional relations (17)–(19), were worked out by the finite difference technique [8]. The steady solution was defined as a solution of the time-dependent problem. A discretization of the convective members was performed by Samarskii monotonic scheme [28] and for the diffusive members the central differences were employed. The parabolic Equations (14)–(16) were worked out using the Samarskii locally one-dimensional scheme [28]. The obtained set of linear equations was worked out by the Thomas algorithm. The stream function equation was approximated using the central differences for the second derivatives. The received set of linear equations was worked out by the successive over relaxation technique. The computations were stopped when the residuals for the stream function get bellow 10^−7^.

For definition of the stream function magnitude at the inner body boundary the special procedure was used [29]. Namely, we introduce the condition that the pressure *p* should be single-valued along the internal block surface. This condition is expressed by:

(22) $$\int \frac{\partial p}{\partial \eta }\;d\sigma =0.$$

Here *η* is the unit tangential vector along the boundary, *σ* is the internal block surface.

**Figure 2:**
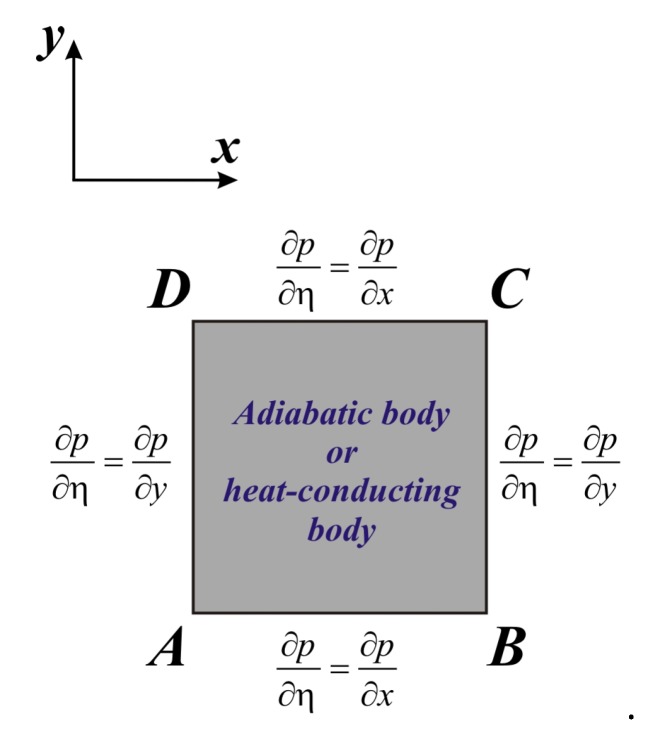
Surface of the internal body.

Taking into account the considered domain of interest (Figure 1) we can define the internal block surface presented in Figure 2:

Using governing Equations (2) and (3) in non-dimensional form as well as non-slip boundary conditions for velocity at internal block surface, ∂p/∂η can be defined as:

(23) $$\frac{\partial p}{\partial x}=\frac{\mu_{nf}}{\mu_{f}}\frac{\rho_{f}}{\rho_{nf}}\sqrt{\frac{Pr}{Ra}}\frac{\partial^{2}u}{\partial y^{2}}$$

(24) $$\frac{\partial p}{\partial y}=\frac{\mu_{nf}}{\mu_{f}}\frac{\rho_{f}}{\rho_{nf}}\sqrt{\frac{Pr}{Ra}}\frac{\partial^{2}v}{\partial x^{2}}+\frac{{\left(\rho \beta \right)}_{nf}}{\rho_{nf}\beta_{f}}\theta .$$

Taking into account the condition (22) we have:

(25) $$\int_{\sigma }dp=\int_{A}^{B}\frac{\partial p}{\partial x}\;dx+\int_{B}^{C}\frac{\partial p}{\partial y}\;dy+\int_{C}^{D}\frac{\partial p}{\partial x}\;dx+\int_{D}^{A}\frac{\partial p}{\partial y}\;dy=0.$$

Introducing Equations (23) and (24) in (25) we have:

(26) $$\int_{A}^{B}\frac{\partial \omega }{\partial y}\;dx-\int_{B}^{C}\frac{\partial \omega }{\partial x}\;dy+\int_{C}^{D}\frac{\partial \omega }{\partial y}\;dx-\int_{D}^{A}\frac{\partial \omega }{\partial x}\;dy=\frac{{\left(\rho \beta \right)}_{nf}}{{\left(\rho \beta \right)}_{f}}\frac{\mu_{f}}{\mu_{nf}}\sqrt{\frac{Ra}{\mathrm{Pr}}}\left(\int_{B}^{C}\theta \;dy+\int_{D}^{A}\theta \;dy\right).$$

Using Equation (26) and interpreting the correlation between vorticity at internal body surface and stream function, the considered value can be found. Such technique was used in the present study for determining the stream function magnitude at inner body boundary.

## 4. Validation

**Figure 3:**
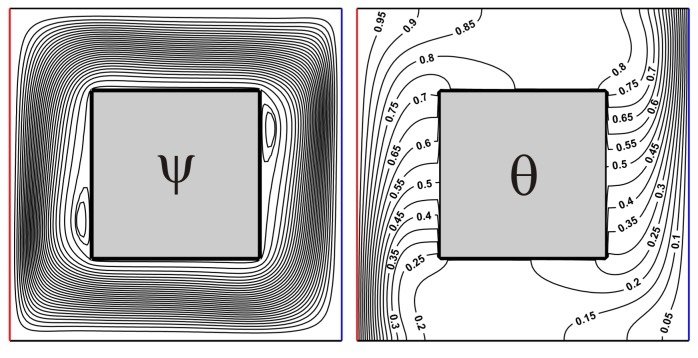
Isolines of ψ and θ for *Ra* = 10^5^, *δ* = 0.5.

**Figure 4:**
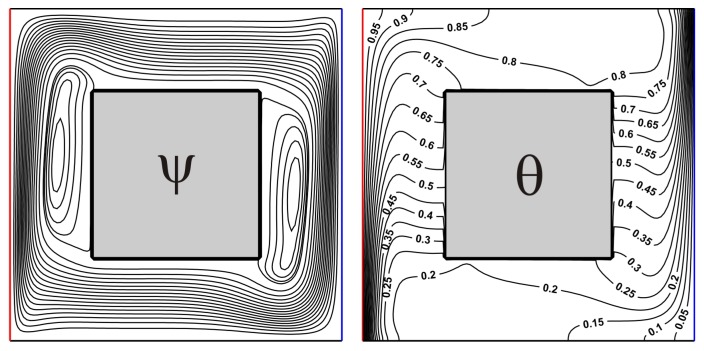
Isolines of ψ and θ for *Ra* = 10^6^, *δ* = 0.5.

The created numerical code was verified employing the numerical results of Karki et al. [30] for convective heat transfer in a square cavity with isothermal vertical walls and centered thermally-insulated body. Figure 3 and Figure 4 demonstrate a good concordance for considered isolines for different *Ra* in comparison with numerical data of Karki et al. [30].

**Figure 5:**
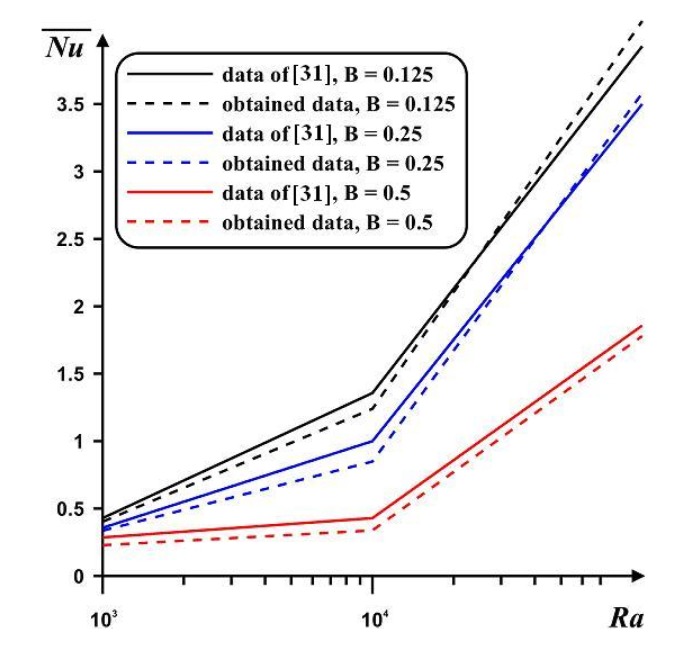
Mean *Nu* at hot border compared with computations [31] for different heights of the obstacles (B) and Rayleigh numbers.

The second benchmark problem was free convective energy transport in a rectangular chamber with two bottom border-mounted adiabatic blocks [31]. Figure 5 presents a very good concordance for the average *Nu* at the hot boundary compared with data of Ben-Nakhi and Chamkha [31].

The third benchmark problem was free convective energy transport of Al_2_O_3_-H_2_O nanoliquid inside a differentially-heated chamber. Table 1 illustrates a very good concordance for the mean *Nu* at heated wall, in dependence on the nanoadditives concentration, in comparison with experimental results [27].

**Figure 6:**
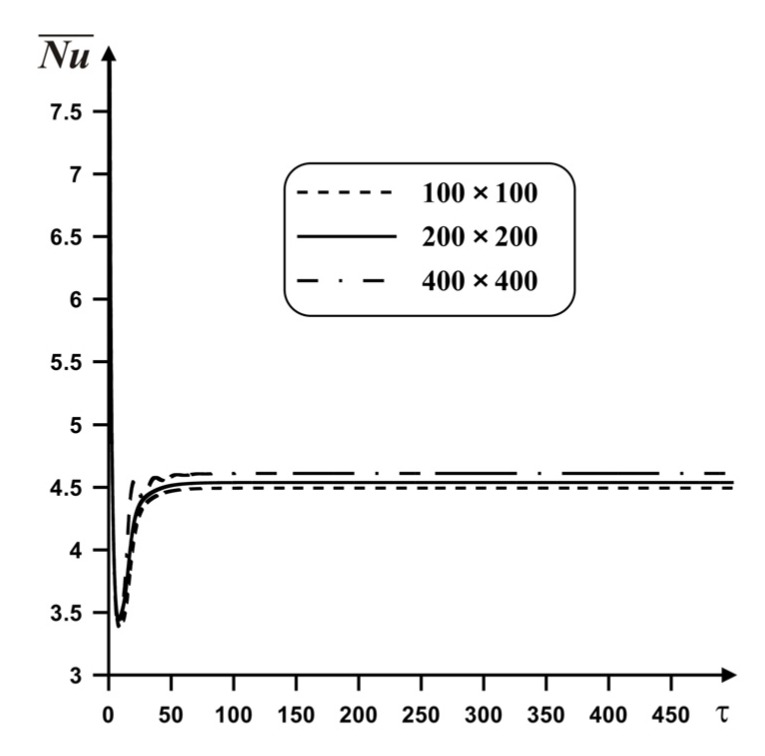
Time profiles of average *Nu* for various mesh parameters.

A mesh independence test was performed employing four different grid parameters (100 × 100, 200 × 200, and 400 × 400) for *Ra* = 10^5^, *Pr* = 6.82, *ϕ* = 0.02, *δ* = 0.5. Using Figure 6 it is possible to conclude that the deviations of the average *Nu* for 200 × 200 and 400 × 400 are negligible (at about 1.5%). Therefore, a uniform grid of 200 × 200 was used for investigations.

## 5. Results and Discussion

**Figure 7:**
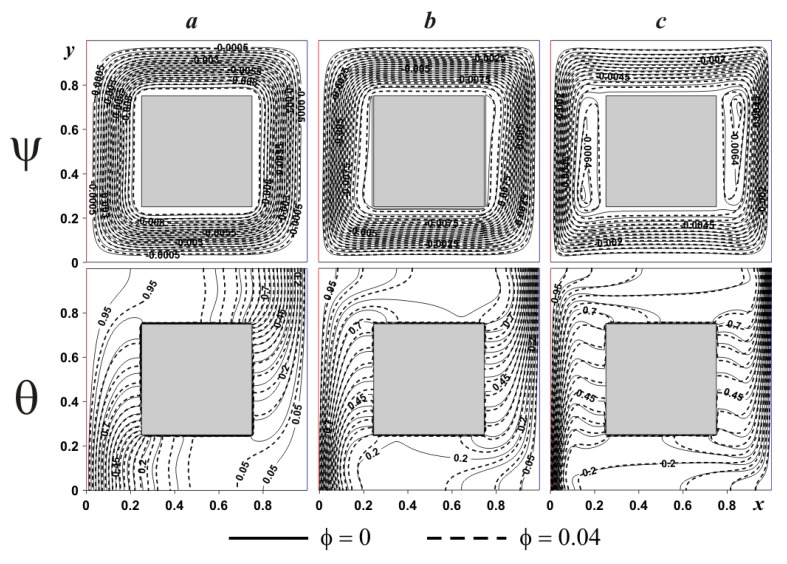
Isolines of ψ and θ for adiabatic internal body at *δ* = 0.5 and different *Ra* and *ϕ*:(**a**)—*Ra* = 10^4^, (**b**)—*Ra* = 10^5^, (**c**)—*Ra* = 10^6^.

**Figure 8:**
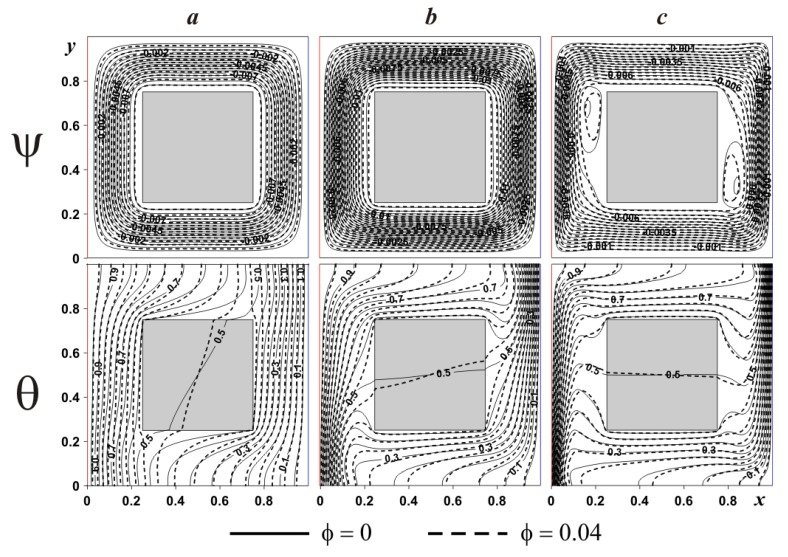
Isolines of ψ and θ for heat-conducting internal body at *δ* = 0.5 and different *Ra* and *ϕ*: (**a**)—*Ra* = 10^4^, (**b**)—*Ra* = 10^5^, (**c**)—*Ra* = 10^6^.

**Figure 9:**
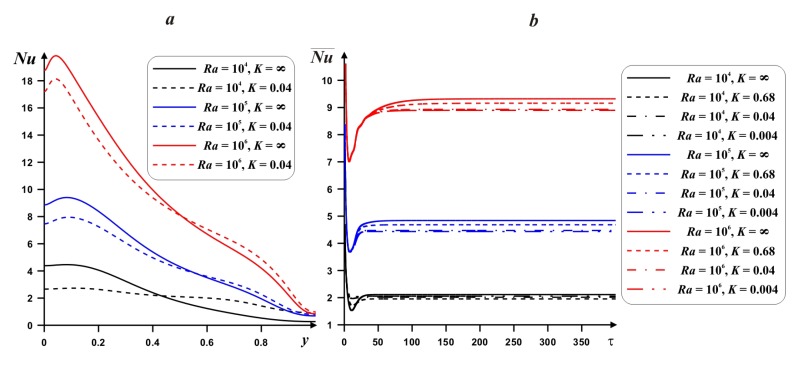
Local *Nu* profiles (**a**) and time-dependent mean *Nu* profiles (**b**) for *δ* = 0.5, *ϕ* = 0.02 and different *Ra* and *K*.

**Figure 10:**
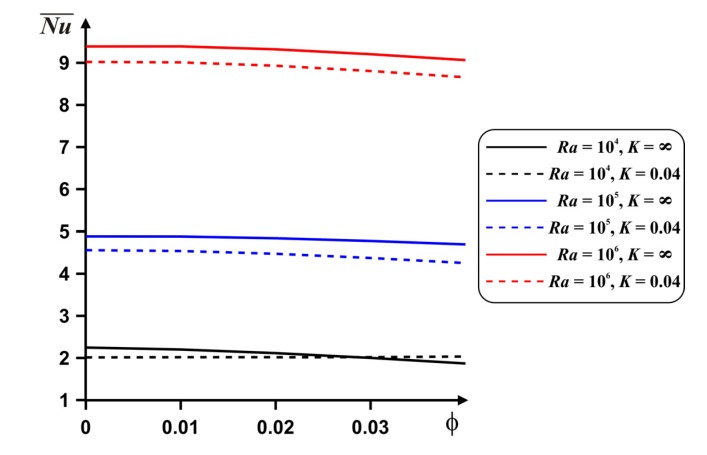
Dependences of the mean *Nu* on *ϕ*, *Ra* and *K* for *δ* = 0.5.

**Figure 11:**
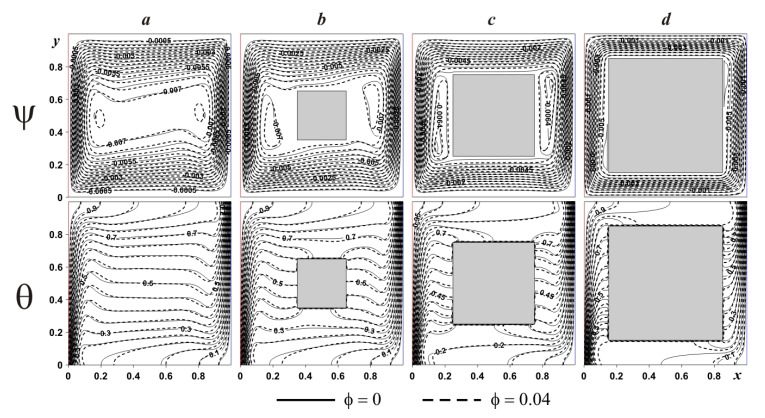
Isolines of ψ and θ for adiabatic internal body at *Ra* = 10^6^ and different *δ* and *ϕ*: (**a**)—*δ* = 0.0, (**b**)—*δ* = 0.3, (**c**)—*δ* = 0.5, (**d**)—*δ* = 0.7.

**Figure 12:**
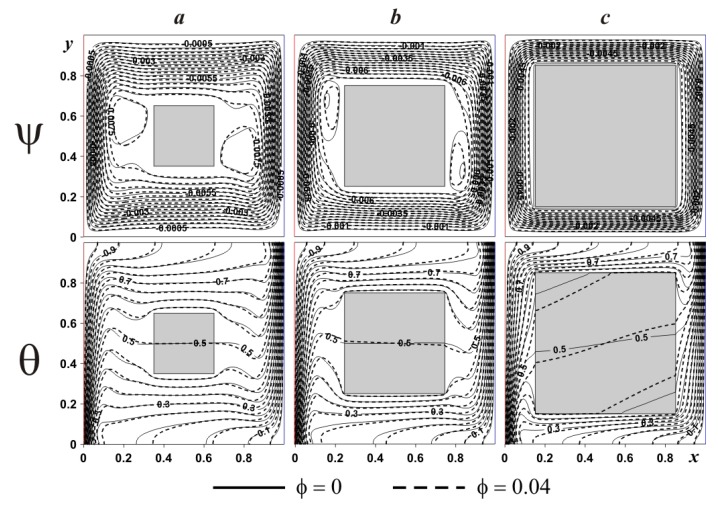
Isolines of ψ and θ for heat-conducting internal body at *Ra* = 10^6^ and different *δ* and *ϕ*: (**a**)—*δ* = 0.3, (**b**)—*δ* = 0.5, (**c**)—*δ* = 0.7.

**Figure 13:**
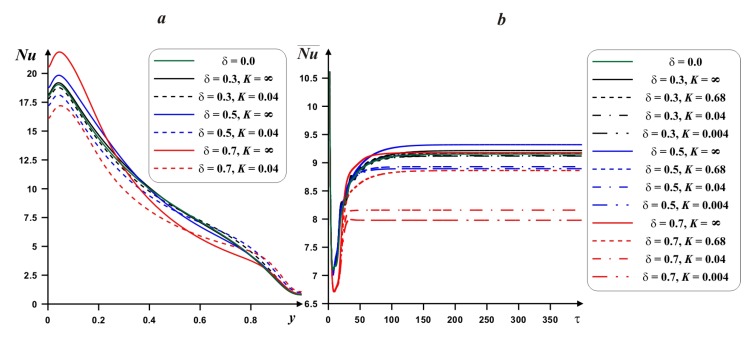
Local *Nu* profiles (**a**) and time-dependent mean *Nu* profiles (**b**) for *Ra* = 10^6^, *ϕ* = 0.02 and different *δ* and *K*.

**Figure 14:**
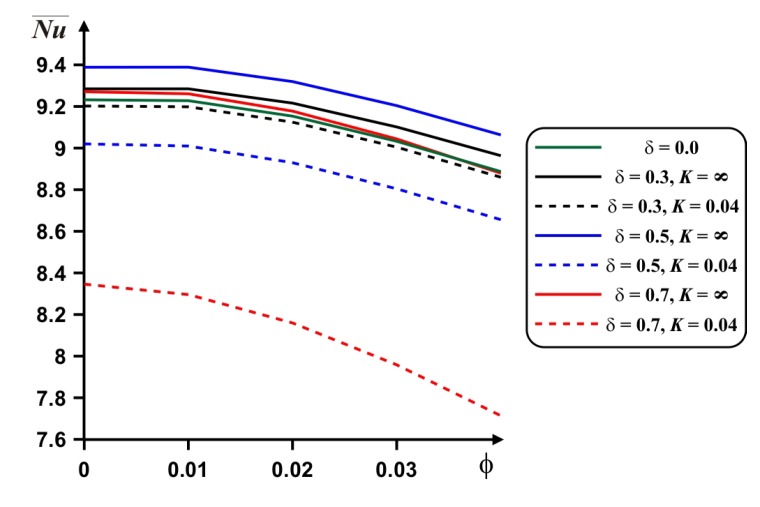
Dependences of the mean *Nu* on *ϕ*, *δ* and *K* for *Ra* = 10^6^.

**Figure 15:**
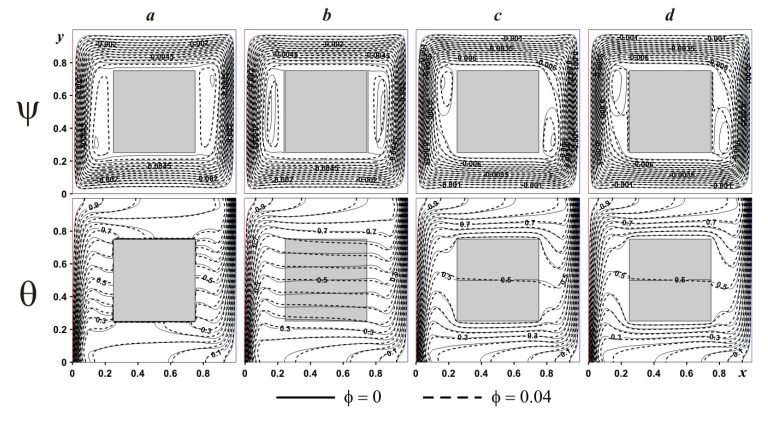
Isolines of ψ and θ for heat-conducting internal body at *Ra* = 10^6^ and different *K* and *ϕ*: (**a**)—*K* = ∞, (**b**)—*K* = 0.68, (**c**)—*K* = 0.04, (**d**)—*K* = 0.004.

Computational investigations have been conducted for Rayleigh number (*Ra* = 10^4^–10^6^), the nanoadditives concentration (*ϕ* = 0.0–0.04), internal body size (*δ* = 0.3–0.7), and a thermal conductivity ratio (*K* = 10^–3^–1). Effects of the mentioned characteristics on circulation field, temperature field and profiles of *Nu* are presented in Figure 7, Figure 8, Figure 9, Figure 10, Figure 11, Figure 12, Figure 13, Figure 14 and Figure 15.

Figure 7 demonstrates the considered isolines within the cavity having internal adiabatic block for various *Ra*. In the case of small magnitude of the buoyancy force (see Figure 7a) one global circulation can be found reflecting an appearance of upstream flows next to the left hot boundary and downstream circulations at the right cold boundary. Temperature field illustrates a formation of heat conduction regime over and under the internal block, where temperature isolines are parallel to vertical boundaries. At the same time, isotherms near the isothermal walls characterize low intensive circulation. A rise of *Ra* (see Figure 7b) reflects more intensive liquid motion with an appearance of thin temperature boundary layers near the vertical borders. Isolines of stream function present a formation of weak recirculations zones near the internal block, namely, close to the left bottom corner and close to the right upper corner. This considered heat transfer mode demonstrates less intensive cooling and heating of the cavity from isothermal walls. Further increment of the buoyancy force bulk results in an expansion of secondary vortices close to the internal body surface. Such circulations reflect the temperature stratification in these zones, where heating occurs from the top portion till the lower part. An addition of nanoadditives (*ϕ* = 0.04) leads to a reduction of liquid circulation strength, while more essential difference in isotherms can be found for low values of *Ra* (see Figure 7a). As a result an inclusion of nanoparticles allows enhancing the heat conduction regime.

In the case of internal heat-conducting solid body (see Figure 8) flow structures and temperature patterns are changed. Regardless of the Rayleigh number the global circulation formed within the chamber is the same like presented in Figure 7, but the temperature field and secondary vortices have another structure. Thus, isotherms characterize the temperature change within the internal solid block from the left and right sides, where internal isotherm (θ = 0.5) is almost parallel to vertical walls.

In the case of *Ra* = 10^5^ isotherms within the internal solid body become parallel to horizontal walls illustrating heating from the top till bottom, while the cooling process occurs in the opposite direction. Also, a stronger circulation characterizes a diminution of the boundary layers thickness, due to the interaction between the hot and cold fluxes. It is interesting to note that a consideration of internal heat-conducting body characterizes a vanishing of the weak recirculations near the block surface for *Ra* = 10^5^ mentioned for adiabatic case and for *Ra* = 10^6^ these vortices have another shape and location of cells. It is worth noting that strong upstream flow close to the left border and a strong downstream one near the right boundary lead to formation of clockwise circulations far from the origin of these flows. An absence of such strong flows in the case of adiabatic block allows forming vortices elongate along the vertical walls of the internal block.

Figure 9 demonstrates the behavior of the local and mean *Nu* at hot border with *Ra*, thermal conductivity ratio and time. An increment of *Ra* characterizes a raise of the local *Nu*. An interaction of the hot and cold heat fluxes near the lower portion of the left border reflects a presence of high *Nu* values in this zone. A rise of *y*-coordinate illustrates a decrease of *Nu*. Moreover, with *Ra* the maximum value of *Nu* approaches the left boundary. Value *K* = ∞ reflects the presence of internal adiabatic body. A growth of the heat conductivity of internal body material characterizes a diminution of the temperature drop in the lower portion and a rise of this temperature difference in the upper zone. The time dependence of the average Nusselt number (Figure 9b) demonstrates a fast reaching the steady state value. An increment of *Ra* reflects a rise of time for the steady state. A diminution of the heat conductivity ratio from adiabatic case (*K* = ∞) till high internal body material thermal conductivity reflects a reduction of the mean *Nu*. Therefore, in the case of adiabatic internal body one can reveal the maximum energy transport strength.

A reduction in the mean *Nu* with nanoadditives is presented in Figure 10. It is possible to intensify the energy transport with *ϕ* only for low *Ra*, where heat conduction is a dominated energy transport mode. Also, only for this regime, the heat transfer enhancement can be found with a rise of the internal body material thermal conductivity for the case of high nanoparticles concentration.

Effect of the internal body size on streamlines and isotherms for adiabatic and heat-conducting blocks is presented in Figure 11 and Figure 12. In the case of adiabatic internal block (Figure 11), an increment of *δ* results in the attenuation of convective motion with a formation of secondary circulations near the internal body surface. A growth of the internal block size from *δ* = 0.3 till *δ* = 0.5 characterizes an elongation of secondary vortices near the solid block surface, while for *δ* = 0.7 these vortices are decreased essentially. At the same time, an increment of *δ* reflects a vertical displacement of the left eddy core in negative *y*-coordinate direction, while the right eddy core displaces vertically in positive *y*-coordinate direction. Temperature fields illustrate the thermal stratification. Moreover, the presence of the adiabatic body divides the temperature isolines into two parts and these two parts are similar to the original isotherms without a solid body. The addition of nanoparticles reflects a modification of temperature field near the horizontal walls, while streamlines are differed in the zone of secondary vortices.

In the case of internal heat-conducting block (see Figure 12), an introduction of solid block characterizes also a rise in size of two secondary vortices but in the top portion of the left body boundary and near the bottom portion of the right body boundary. The reason for such a difference between adiabatic and heat-conducting bodies was discussed above. Moreover, for *δ* ≤ 0.5 heating/cooling of the internal block occurs in vertical direction, while for *δ* = 0.7, temperature profiles can be found within the solid block that are not parallel to horizontal walls. The addition of nanoparticles reduces the convective flow strength due to a growth of nanofluid viscosity (see Equation (11)). Significant difference in isotherms can be found within the solid body due to different heat fluxes at solid body surface.

Effect of internal block size and heat conductivity ratio on *Nu* and $\bar{Nu}$ is demonstrated in Figure 13. As it has been described above, a rise of the heat conductivity of solid block material results in a diminution of the mean *Nu*. At the same time, the growth of the solid block size illustrates a rise of the temperature drop at the lower portion of the heated wall and an increment of the internal block material thermal conductivity results in strong diminution of local *Nu* at the lower portion of left border. The internal body length has a non-monotonic impact on the mean *Nu*. Therefore, it is possible to reveal an optimal magnitude of *δ* for high value of $\bar{Nu}$. At the same time, a rise of *δ* characterizes more essential impact of *K* on the mean *Nu*.

Diminution of the mean *Nu* with nanoadditives concentration is presented in Figure 14. More essential reduction can be found for high value of the internal block material thermal conductivity. In this case for *δ* = 0.5 we have maximum $\bar{Nu}$.

An impact of the heat conductivity ratio on the considered isolines is shown in Figure 15. A reduction of this parameter illustrates modification of temperature field, where one can reveal a rise of the temperature wave speed within the solid block and as a result a density of isotherms rises near the solid block surface. At the same time, a reduction and displacement of the secondary convective cells cores occur with a decrement of *K*.

## 6. Conclusions

Numerical investigation of thermogravitational convection inside a differentially-heated domain with inner adiabatic or thermally-conducting block has been performed. Investigations have been carried out using the developed computational code based on the non-primitive variables. The detailed description of the numerical procedure for the determining the stream function magnitude the inner body surface has been provided. It should be noted that using the non-primitive variables decreases the number of governing equations due to an exclusion of the pressure field from the momentum equation. Such an approach results in the reduction of computational time. Therefore, using the non-primitive variables allows reducing computational time for various problems. In the case of present study the effective numerical algorithm has been developed for the multi-connected domains. This algorithm has been employed to investigate conjugate natural convection inside the differentially-heated chamber filled with a nanosuspension.

As a result, an analysis of the Rayleigh number, thermal conductivity ratio, internal block size, and nano-additives concentrations has been conducted. It has been ascertained that:

- a decrease in heat conductivity ratio from adiabatic case until high internal body material thermal conductivity illustrates a reduction of the mean *Nu*;
- an internal block size has a non-monotonic impact on the mean *Nu*. Therefore, it is possible to reveal an optimal value of *δ* (0.5 for the present analysis) for high value of $\bar{Nu}$. A rise of *δ* illustrates stronger impact of *K* on $\bar{Nu}$;
- an increment of the nanoadditives concentration characterizes the energy transport degradation, while it is possible to enhance the energy transport with *ϕ* only for low *Ra*, where thermal conduction is a dominated thermal transmission mode.

The performed analysis allows intensifying the heat transfer within the chamber in the case of optimal selection of the inner block size, thermal conductivity of this block material and concentration of nano-sized additives within the host fluid.

The developed numerical algorithm and computational code will be used in future for analysis of the cooling effect in the case of heat-generating internal solid block that can be considered an electronic chip within the electronic cabinet. Moreover, the developed computational technique will be used for an investigation of the effects of several internal heat-generating blocks in order to optimize the location of such an element inside the electronic cabinet. The mentioned analysis will be conducted in the case of a single or hybrid nanoliquid impact.

## Figures and Tables

**Table 1:** Values of mean *Nu* at hot border.

*φ*	*Ra*	*Pr*	Experimental Data [27]	Present Study
0.01	7.74547 × 10^7^	7.0659	32.2037	30.6533
0.02	6.6751180 × 10^7^	7.3593	31.0905	30.5038
0.03	5.6020687 × 10^7^	7.8353	29.0769	30.2157

## Nomenclature

*c*	heat capacity
*g*	gravity force acceleration
*K*	heat conductivity ratio
*k*	heat conductivity
*L*	length and height of the chamber
*Nu*	local Nusselt number
$\bar{Nu}$	average Nusselt number
*p*	non-dimensional pressure
$\bar{p}$	dimensional pressure
*Pr*	Prandtl number
*Ra*	Rayleigh number
*T*	dimensional temperature
*t*	dimensional time
*T_c_*	cold boundary temperature
*T_h_*	hot boundary temperature
*u*, *v*	non-dimensional velocity projections
$\bar{u},\;\bar{v}$	dimensional velocity projections
*V*_0_	dimensional reference velocity
*x*, *y*	non-dimensional Cartesian coordinate
$\bar{x},\;\bar{y}$	dimensional Cartesian coordinates
**Greek symbols**	
*β*	heat expansion parameter
*δ*	non-dimensional size of the internal solid block
*θ*	non-dimensional temperature
*μ*	dynamic viscosity
*ρ*	density
*ρc*	thermal capacitance
*ρβ*	buoyancy parameter
*τ*	non-dimensional time
*γ*	non-dimensional magnitude of the stream function at inner body borders
*ϕ*	nano-sized particles volume fraction
$\bar{\psi }$	stream function
*ψ*	non-dimensional stream function
$\bar{\omega }$	dimensional vorticity
*ω*	non-dimensional vorticity
**Subscripts**	
*c*	cold
*f*	fluid
*h*	hot
*nf*	nanofluid
*p*	(nano)particle
*s*	solid
